# Efficacy of *Bacillus coagulans* BC01 on loperamide hydrochloride-induced constipation model in Kunming mice

**DOI:** 10.3389/fnut.2022.964257

**Published:** 2022-09-21

**Authors:** Xu Zhou, Yafang Chen, Xin Ma, Yang Yu, Xueping Yu, Xiaoyong Chen, Huayi Suo

**Affiliations:** ^1^College of Food Science, Southwest University, Chongqing, China; ^2^The First People's Hospital of Kunshan, Suzhou, China; ^3^Thankcome Biological Science and Technology Suzhou Co., Ltd., Suzhou, China

**Keywords:** *Bacillus coagulans*, constipation, loperamide hydrochloride, intestinal function, fecal microbial diversity

## Abstract

In this study, the laxative effect of *Bacillus coagulans* BC01 (BC01) in mice was investigated using a functional constipation mouse model. Six-week-old male specific pathogen-free (SPF) Kunming mice were randomly divided into five groups: normal control group (saline), model group (loperamide hydrochloride), drug control group (bisacodyl), BC01 low-dose group (4.0 × 10^8^ CFU/mL) and BC01 high-dose group (4.0 × 10^9^ CFU/mL). Except for the normal group, the functional constipation model was established by administering 0.25 mL of a loperamide hydrochloride suspension (1 mg/mL) twice daily for four consecutive days by oral gavage. After modeling, the BC01 groups were administered 0.25 mL of BC01. The bisacodyl served as a control and was administered orally at a dose of 100 mg/kg, while the other groups were administered 0.25 mL of sterile saline. After 7 days of continuous administration, the experimental mice were again induced by loperamide hydrochloride. During this period, the mechanism of BC01 to improve constipation symptoms in mice was analyzed by measuring the changes in body weight, fecal water content, small intestine propulsion rate, histology of small intestinal tissue sections, fecal microbial diversity, serum indices, as well as mRNA and protein expression levels in the small intestinal tissue. BC01 was found to significantly promote the intestinal propulsion rate and increase the fecal water content in the mice. BC01 could also alleviates constipation by regulating gastrointestinal motility (substance P, motilin, endothelin-1, somatostatin, and vasoactive intestinal peptide), gene expression (*c-Kit, SCF, COX-2, NF-*κ*B, iNOS*, and *eNOS*), intestinal inflammation (eNOS, iNOS, NF-κB), and the intestinal microbiota composition in the constipated mice. In addition, the high-dose BC01 treatment had the best preventive effect on constipation. BC01 is a probiotic strain to effectively relieve the adverse effects of constipation.

## Introduction

Constipation is a clinically common digestive dysfunction characterized by persistent, infrequent, or incomplete bowel movements (1). Due to changes in eating habits and increased life stress levels, among other changes in lifestyle habits, constipation symptoms are increasingly being observed in young people all throughout the world (2). Constipation causes a large amount of excrement to accumulate in the intestinal tract, where harmful bacteria could accumulate and may produce toxic substances. These toxic substances can be reabsorbed by the colon and circulate through the body, which has long term effects on intestinal function and human health (3). Chronic constipation can engender obesity, enteritis, and even colorectal cancer. Constipation-derived diseases increase the physical, economic, and social burden of patients and place additional strain on the healthcare system. Although constipation is commonly treated with increased exercise and dietary fiber intake, various laxatives are currently available for severe cases to relieve the symptoms. While laxatives generally work quickly, constipation symptoms often return or worsen once the medication is stopped. However, while there are tremendous challenges when developing new treatment methods to effectively prevent constipation, the development of effective treatments that can reduce the impact of the constipation on the lifestyles of patients are incredibly important.

Intestinal microbes are responsible for maintaining a dynamic homeostatic balance within the colon and have a regulatory effect on the health of host (4, 5). Patients suffering from constipation have imbalances in their intestinal species, including decreased dominant species and increased harmful microbiota relative to healthy people (5–7). Previous data on the intestinal microbiota structure of patients with chronic constipation showed a higher number of conditioned pathogens and a lower number of obligate anaerobic bacteria compared to healthy individuals (8). These differences may affect the change in the local metabolic environment of the intestine and the ability to exercise (9). Therefore, repairing the gut microenvironment by introducing healthy gut microbiota may alleviate constipation-related symptoms.

*B. coagulans* is a spore-producing probiotic with better tolerance in production, storage, and gastrointestinal conditions compared to non-spore-forming probiotics such as *Bifidobacterium a*nd *Lactobacillus* (10). Toxicological experiments and a large number of clinical observations show that *B. coagulans* is safe, without mutagenicity, teratogenicity, or genotoxicity, and has been widely used in medicine, food, and chemical industries. It has therapeutic effects on intestinal diseases such as acute diarrhea, irritable bowel syndrome, antibiotic-associated diarrhea, constipation, and colitis by modulating microbiota composition, host immunity, and metabolism (11). A clinical trial in Japanese volunteers with functional constipation showed that consumption of *B. coagulans* lilac-01 (1 × 10^8^ CFU per day for 2 weeks) significantly improved defecation and fecal characteristics (12). In adults with mild intermittent constipation and a habitually low intake of fruits and vegetables, *B. coagulans* SNZ 1969 was able to improve intestinal motility and intestinal microbiota composition (13).

*B. coagulans* BC01 (BC01) was isolated from homemade thick broad bean sauce in Sichuan Province, China. It has been archived in the China Center for Type Culture Collection and was numbered CCTCC number M2017813 (14). BC01 has good gastrointestinal tract tolerance. In this study, the effect of BC01 in relieving drug-induced functional constipation was investigated, and its mechanism of action was preliminarily discussed. A functional mice constipation model was established by administering loperamide hydrochloride to specific pathogen-free (SPF) Kunming mice, and the effects of BC01 on improving constipation was analyzed by monitoring the changes in the body weight, fecal water content, intestinal propulsion rate, gut microbiome diversity, and histology of tissue sections of the mice. Then, the mechanism of BC01 on the prevention of constipation were further evaluated by analyzing the serum indices, expression profiles of genes implicated in constipation, and the expression levels of intestinal proteins implicated in inflammation. This study provides an experimental and theoretical basis for the utilization of BC01 as a probiotic in health food and medicine.

## Materials and methods

### Strain preparation

The preserved BC01 bacteria powder was inculated into liquid MRS Broth medium (Beijing Land Bridge Technology Co., Ltd, Beijing, China), incubated at 37°C for 24 h, and three consecutive resuscitations. Inoculate and expand the culture at a proportion of 2% (v/v). Centrifuged the culture medium at 8 000 rpm/min for 10 min at 4°C to removed the supernatant, collected the bacterial mud, and counted the plates. The bacteria were configured with sterile saline to a concentration of 4.0 × 10^8^ CFU/mL and 4.0 × 10^9^ CFU/mL, and the OD600 nm value of the bacterial solution at this concentration was measured and stored at 4°C for later use.

### Drug preparation

A loperamide hydrochloride suspension (1 mg/mL) was prepared by dissolving the contents of 50 capsules of loperamide hydrochloride (2 mg/capsule) in 100 mL of sterile water immediately prior to use. An activated carbon suspension was prepared by adding gum arabic (50 g) in sterile water (400 mL) and boiling the mixture until transparent. Then, the solution was supplemented with activated carbon (25 g), and the suspension was boiled three times. After cooling, the solution was diluted with additional water to 500 mL to obtain a 50 g/L activated carbon suspension, which was stored at 4°C.

### Animal experiments

Six-week-old male SPF Kunming mice (25 ± 5 g) were acquired from Hunan Slike Jingda Laboratory Animal Co., Ltd. [License number: SCXK (Xiang) 2019-0004]. The animals were housed in the Animal Laboratory of Southwest University, which was approved by the Institutional Animal Care and Use Committee of Southwest University of China (IACUC-20211020-06). During the feeding period, mice had free access to water, and the housing facility operated under a 12 h light/12 h dark cycle with a constant humidity (40–50%) and temperature (22–25°C).

After a one-week acclimatization period, 40 mice were randomly divided into 5 groups (8 mice/group): normal control group (NC), model group (M), drug control group (DC), BC01 low-dose group (BC01-L) and BC01 high-dose group (BC01-H). During the experiments, from day 1 to 4, the mice in all groups (except for NC) were administered 0.25 mL of the 1 mg/mL loperamide hydrochloride suspension twice daily by oral gavage to induce the functional constipation model. After completing the gavage on the fourth day, all groups fasted for 16 h. From days 5 to 11, the NC and M groups were administered 0.25 mL of sterilized saline daily, and the BC01-L and BC01-H groups were administered 0.25 mL bacterial suspension of 4.0 × 10^8^ CFU/mL and 4.0 × 10^9^ CFU/mL BC01 daily, respectively. Then, 0.25 mL of a 100 mg/kg bisacodyl water solution was administered to DC by oral gavage daily. After completion of the dosage regimen on the 11th day, the mice in all groups fasted for 16 h. Following, blood serum, tissue samples (small intestine and colon), and feces were collected for analysis. To collect the serum, the blood after withdrawal from orbital vein was refrigerated at 4°C for 2 h and centrifuged at 3,000 rpm/min at 4°C for 10 min, and the serum (top layer) was removed (15). All collected samples were stored in an ultra-low temperature freezer until subsequent analysis.

### Determination of the intestinal propulsion rate

At the end of experiments, mice fasted overnight (16 h) to empty their intestinal contents. Each mouse was orally fed 0.25 mL of the activated charcoal suspension and then sacrificed under anesthesia after 20 min. The abdominal cavity of each mouse was quickly opened, and the entire intestine from the pylorus to anus was dissected. The mouse intestinal propulsion rate was calculated as *D* = *L*2/*L*1 × 100%, where L1 was the full length of the intestine after straightening without tension, and L2 was the movement length of activated carbon in the intestine.

### Determination of the fecal water content

In the morning following daily oral gavage, the animals were placed in a new cage, and before administration of the treatment in the afternoon, the feces were collected and weighed, and the wet weight of feces was recorded as W1. The collected feces were paced in a blower for drying and dehydration at 105°C, and the dry weight was recorded as W2. Finally, the fecal moisture content of mice was calculated as *R* = (*W*1−*W*2)/*W*1 × 100%.

### Hematoxylin and eosin staining

The small intestine was fixed in 4% paraformaldehyde for 24 h and stained with H&E (16). The tissue sections were observed under an upright microscope to determine the pathological morphology of the small intestinal tissue of the constipated mice.

### Determination of gastrointestinal hormone levels in serum

The levels of excitatory gastrointestinal hormones, including substance P (SP) and motilin (MTL), as well as the levels of inhibitory gastrointestinal hormones, including endothelin 1 (ET-1), somatostatin (SS), and vasoactive intestinal peptide (VIP), in the serum of the mice were measured using ELISA kits (Shanghai Enzyme-Linked Biotechnology Co., Ltd., Shanghai, China) according to the manufacturer's instructions.

### Determination of mRNA expression of related genes in small intestinal tissue by real-time quantitative PCR

Six samples were randomly selected from each group for determination of mRNA expression of related genes in small intestinal tissue. The mRNA expression levels of *COX-2, c-Kit, NF-*κ*B, iNOS, eNOS* and *SCF* were quantified by RT-qPCR (Shanghai Yisheng Biotechnology Co., Ltd., Shanghai, China). The total RNA was extracted from small intestinal tissue and reverse-transcribed into cDNA using an RNA extraction kit (Beijing Solarbio Science & Technology Co., Ltd., Beijing, China) and a cDNA reverse transcription kit (Shanghai Yisheng Biotechnology Co., Ltd., Shanghai, China) according to the manufacturers' instructions. A microspectrophotometer was used to determine the purity of the RNA by measuring the ratio of the absorbances at 260 nm and 280 nm. For RT-qPCR analysis, β-actin served as a normalized internal control. The final expression of the RT-qPCR product was calculated using the 2^−Δ*ΔCt*^ method.

### Small intestine tissue protein extraction and Western-Blot analysis

The small intestine samples extracted from the mice were homogenized by centrifugation at 12,000 g for 20 min at 4°C, and the total protein from the homogenized tissue was extracted using a Solarbio Whole Protein Extraction Kit. The total concentration of protein was determined using a BCA Protein Assay Kit (Beijing Solarbio Science & Technology Co., Ltd., Beijing, China). For Western-Blot analysis, 50 μg of the protein extracts were loaded onto a 10% NuPAGE gel and run to isolate the proteins, and then transferred to polyvinylidene fluoride (PVDF) membranes. The PVDF membranes were blocked with 5% skim milk by shaking the membranes at 28°C and 50 rpm for 1 h. Then, the membranes were incubated with primary antibodies (anti-iNOS, anti-eNOS, anti-NF-κB) (Thermo Fisher Scientific Co., Ltd., Shanghai, China) overnight at 4°C. The PVDF membranes were washed 5 times with 1 × TBST for 5 min each, after which they were incubated with HRP-labeled diantibodies (Thermo Fisher Scientific Co., Ltd., Shanghai, China) for 1 h and washed five times with 1 × TBST for 5 min each. Finally, the PVDF membranes were stained with an ECL chemiluminescence solution to observe antibody binding, which was quantified using the ImageJ (National Institutes of Health) software package.

### Fecal microbial diversity analysis

Four samples of feces were randomly selected from each group for intestinal microbiota analysis. Total microbial genomic DNA was extracted from mouse feces samples using the E.Z.N.A.® soil DNA Kit (Omega Bio-tek, Norcross, GA, U.S.) according to manufacturer's instructions. The quality and concentration of DNA were determined by 1.0% agarose gel electrophoresis and a NanoDrop® ND-2000 spectrophotometer (Thermo Scientific Inc., USA) and kept at −80°C prior to further use. The hypervariable region V3-V4 of the bacterial 16S rRNA gene were amplified with primer pairs 338F (5'-ACTCCTACGGGAGGCAGCAG-3') and 806R (5'- GGACTACHVGGGTWTCTAAT-3') (17) by an ABI GeneAmp® 9700 PCR thermocycler (ABI, CA, USA). The PCR reaction mixture including 4 μL 5 × Fast Pfu buffer, 2 μL 2.5 mM dNTPs, 0.8 μL Forward Primer (5 μM), 0.8 μL Reverse Primer (5 μM), 0.4 μL Fast Pfu polymerase, 10 ng of template DNA, 0.2 μLBSA and ddH_2_O to a final volume of 20 μL. PCR amplification cycling conditions were as follows: initial denaturation at 95°C for 3 min, followed by 27 cycles of denaturation at 95°C for 30 s, annealing at 55°C for 30 s and extension at 72°C for 45 s, and single extension at 72°C for 10 min, and 10°C until halted by user. All samples were amplified in triplicate. The PCR product was extracted from 2% agarose gel and purified using the AxyPrep DNA Gel Extraction Kit (Axygen Biosciences, Union City, CA, USA) according to manufacturer's instructions and quantified using Quantus™ Fluorometer (Promega, USA).

Purified amplicons were pooled in equimolar amounts and paired-end sequenced on an Illumina NovaSeq PE250 platform (Illumina, San Diego, USA) according to the standard protocols by Majorbio Bio-Pharm Technology Co. Ltd. (Shanghai, China). The raw sequencing reads were deposited into the NCBI Sequence Read Archive (SRA) database (Accession Number: PR JNA 848956).

### Data analysis

GraphPad 7.0 was employed for graphing the quantitative data acquired from each experiment. All data were analyzed for statistical significance using SPSS 20.0, and one-way analysis of variance (ANOVA) was used to compare the statistical differences among groups. All data were reported as the mean ± SD, and *p* < 0.05 indicated a significant difference.

## Results

### Effects of BC01 on body weights of mice

**Figure 1:**
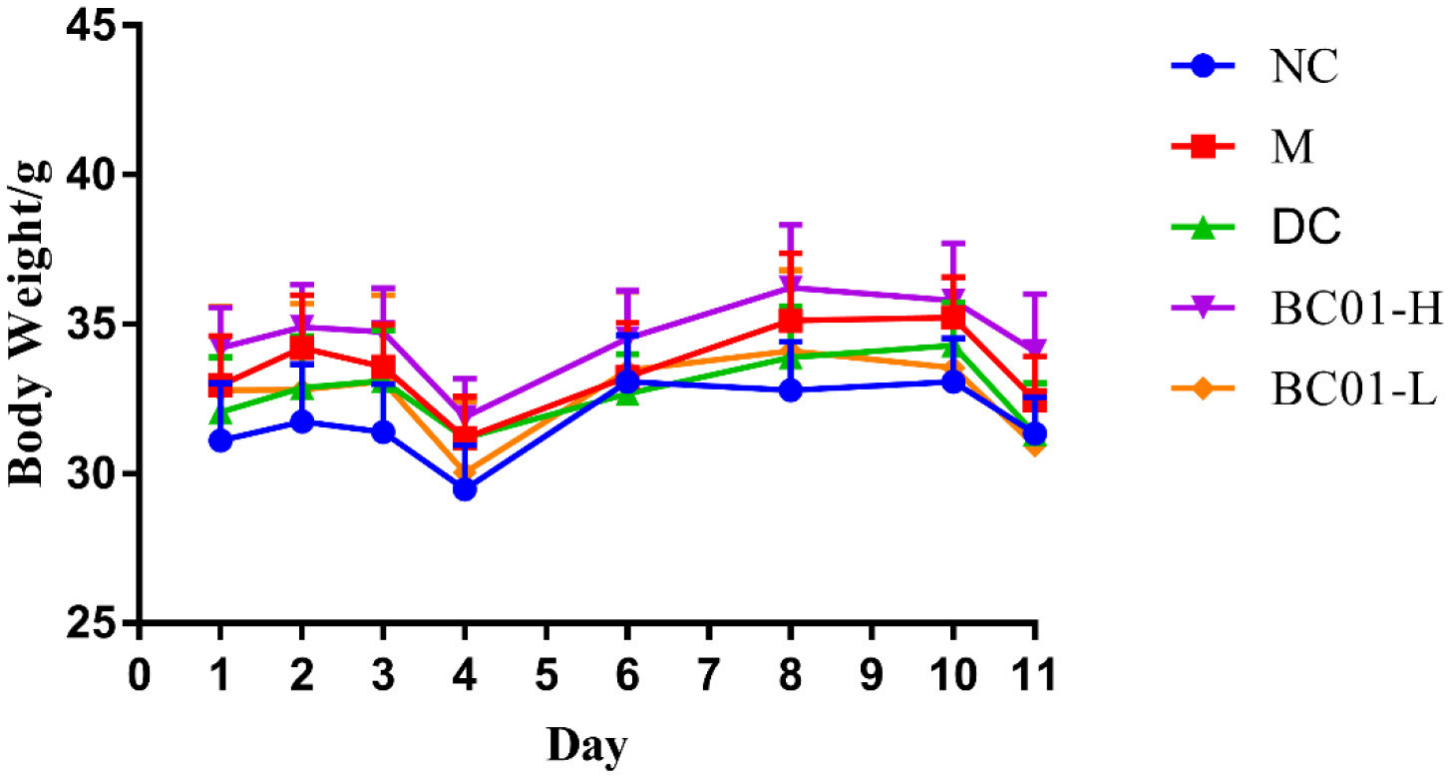
Changes in the body weights of mice. NC, normal group; M, constipation model group; DC, bisacodyl control group; BC01-H, *B. coagulans* BC01 high-dose group; BC01-L, *B. coagulans* BC01 low-dose group (*n* = 8).

The changes in the body weights of the mice during the experiments are shown in Figure 1. During the experiment, the body weights of the mice in each group increased. During the modeling period, the body weights of the mice tended to increase and then decrease, and significantly decreased on the last day of modeling (*p* < 0.05). However, there was no significant difference in body weight between the groups (*p* > 0.05). After treatment, all mice regained weight. However, interestingly, the mice lost body weights during the later stages of the experiment (*p* < 0.05), but there was also no significant difference in body weight between the groups, which indicated that treatment might alleviate the body weights gain of constipated mice to a certain extent. BC01 has no significant effect on the body weight of mice.

### Effects of BC01 on fecal moisture content in the constipated mice

**Figure 2:**
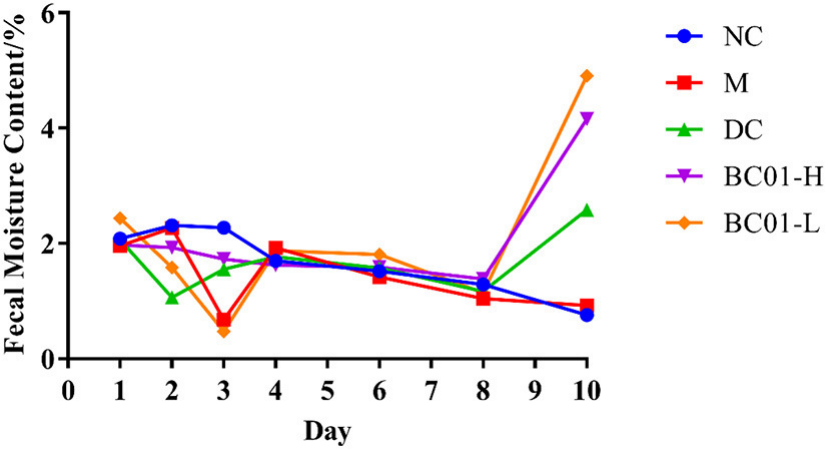
Changes of fecal moisture content in mice. NC, normal group; M, constipation model group; DC, bisacodyl control group; BC01-H, *B. coagulans* BC01 high-dose group; BC01-L, *B. coagulans* BC01 low-dose group (*n* = 8).

The constipated mice exhibited lower fecal water content, dryer stool, and weakened intestinal peristalsis function compared to the control mice. As shown in Figure 2, during the modeling period, the fecal water contents in all groups decreased to varying degrees, except for the NC group. After the intervention treatment, the fecal water contents of the mice fed with BC01 increased compared to M, with the fecal water contents in the BC01-fed mice being higher than in the DC group. These results showed that BC01 could relieve constipation symptoms by increasing the fecal water content in the intestines of the constipated mice.

### Effects of BC01 on small intestinal propulsion rate in the constipated mice

**Figure 3:**
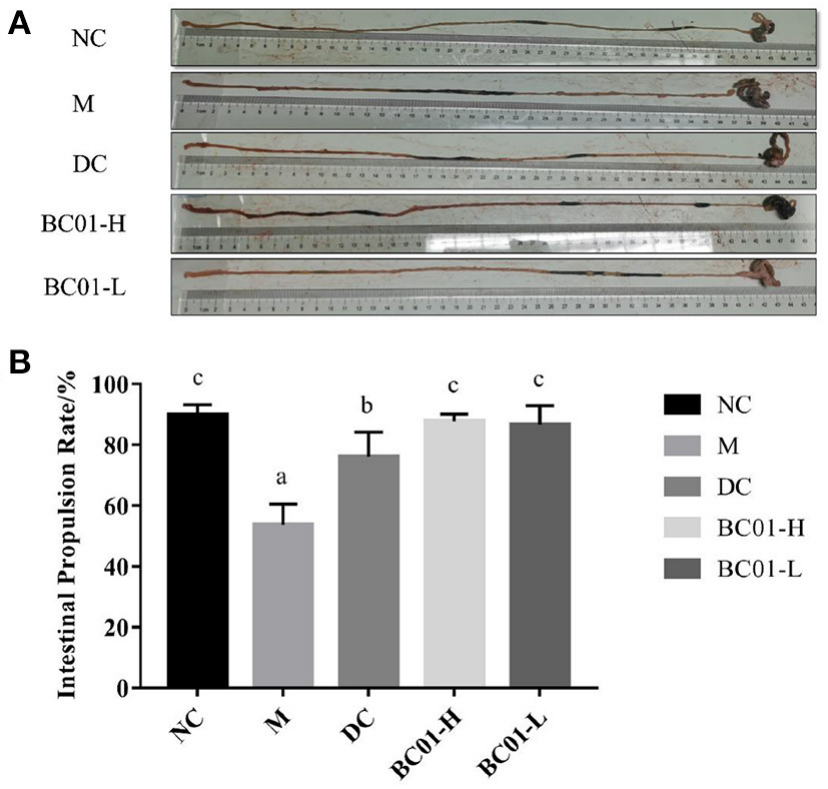
Small intestinal propulsion rate in mice. **(A)** Small intestine anatomy. **(B)** Small bowel advancement rate. The different letters among the experimental groups indicated statistical differences (*p* < 0.05). NC, normal group; M, constipation model group; DC, bisacodyl control group; BC01-H, *B. coagulans* BC01 high-dose group; BC01-L, *B. coagulans* BC01 low-dose group (*n* = 8).

The changes in the intestinal propulsion rates are shown in Figure 3. In the M group, the intestinal propulsion rate was 53.73 ± 6.33%, while the intestinal propulsion rate in the NC group was 89.75 ± 3.26%. Therefore, the intestinal propulsion rate was significantly higher in the mice in the NC group compared to in the M group (*p* < 0.05). After the treatment intervention, the rate of intestinal propulsion showed a statistically significant improvement (*p* < 0.05) in BC01 group. For example, the intestinal propulsion rate in the BC01-H group was 87.78 ± 2.19%, which was 34.06 ± 8.50% higher than the rate in the M group. These results indicated that BC01 could accelerate the movement of the activated carbon through the small intestine and promote peristalsis, thereby improving constipation.

### Effects of BC01 on the histomorphology of the small intestine

**Figure 4:**
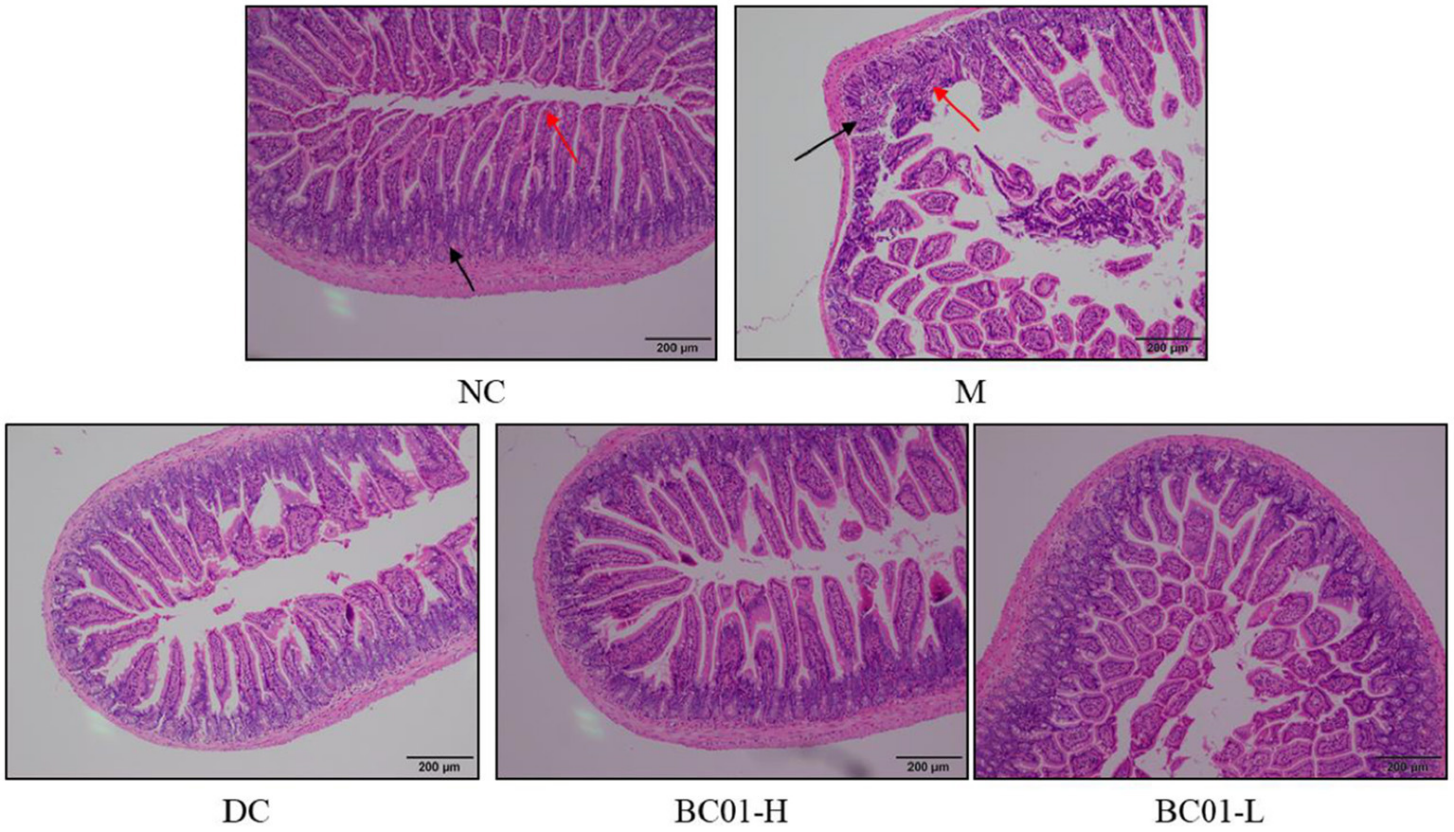
Histology of the small intestine tissue sections stained with H&E stain. NC, normal group; M, constipation model group; DC, bisacodyl control group; BC01-H, *B. coagulans* BC01 high-dose group; BC01-L, *B. coagulans* BC01 low-dose group.

The microscopic images enlighten that in Figure 4 that the NC group had normal intestinal tissue structure; the mucosal layer villi were long, neatly and closely arranged; the number was not reduced, and the epithelial cells were not significantly degenerated and shed, as shown by the red arrow; the crypt tissue would be better, as shown by the black arrow. However, the intestinal tissue structure in the M group was abnormal. The number of villi in some areas of the mucosal layer decreased, the residual villi became shorter than those in the NC group, the epithelial cells eroded and fell off, and the lamina propria was exposed, as shown by the red arrow. The number of crypts decreased, and inflammatory cells and fibrous tissue hyperplasia can be seen, as shown by the black arrow. After treatment, the intestinal tissue structure was basically normal, the villi of the mucosal layer were arranged neatly and closely, and the number did not decrease. There was no obvious degeneration and abscission of epithelial cells, and there was no obvious inflammatory cell infiltration in the tissue.

### Effects of BC01 on levels of gastrointestinal-related hormones in the mice serum

As shown in Table 1, the levels of SP and MTL in the sera of the mice in the M group were significant lower than in the NC group (*p* < 0.05), while the levels of ET-1, SS, and VIP were significant higher (*p* < 0.05). After treatment, the levels of SP in the serum of the BC01-fed mice had dramatically improved (*p* < 0.05) compared to the mice in the M group, while the levels of ET-1, SS, and VIP were significantly lower. These results indicated that BC01 could accelerate the gastrointestinal transport and relieve constipation by regulating the secretion levels of excitatory gastrointestinal hormones and inhibitory gastrointestinal hormones.

**Table 1:** Serum indicators.

	**MTL** ** (pg/mL)**	**SP** ** (pg/mL)**	**ET-1** ** (pg/mL)**	**SS** ** (pg/mL)**	**VIP** ** (pg/mL)**
NC	68.12 ± 2.71^b^	54.93 ± 2.39^bc^	20.69 ± 0.92^a^	26.88 ± 0.85^a^	34.21 ± 1.30^a^
M	59.51 ± 1.71^a^	49.44 ± 3.93^a^	24.07 ± 1.64^b^	30.06 ± 1.64^b^	37.91 ± 2.15^b^
DC	60.73 ± 0.37^a^	51.60 ± 3.48^ab^	21.44 ± 1.28^a^	28.09 ± 1.89^a^	36.13 ± 2.37^ab^
BC01-H	60.50 ± 1.61^a^	57.32 ± 6.38^c^	23.80 ± 1.85^b^	27.52 ± 0.85^a^	33.97 ± 3.93^a^
BC01-L	59.65 ± 1.96^a^	57.91 ± 3.28^c^	23.81 ± 1.99^b^	28.46 ± 1.66^a^	35.19 ± 2.47^ab^

NC, normal group; M, constipation model group; DC, bisacodyl control group; BC01-H, B. coagulans BC01 high-dose group; BC01-L, B. coagulans BC01 low-dose group (*n* = 8); ET-1, endothelin 1; MTL, motilin; SP, substance P; SS, somatostatin; VIP, vasoactive intestinal peptide. ^a−c^Means in the same column with different lowercase superscript letters are significantly different at *P* < 0.05.

### Effects of BC01 on mRNA expression

The mRNA expression levels of *COX-2, eNOS, iNOS, c-Kit, SCF*, and *NF-*κ*B* in the small intestinal tissues of constipated mice were measured by RT-qPCR, and the results are shown in Table 2. The expression levels of *COX-2, NF-*κ*B*, and *iNOS* were all higher in the small intestinal tissue of the mice in BC01 group compared to the mice in the M group, while the expression levels of *c-Kit, SCF, eNOS* were all lower. Compared to the M group, the mRNA expression of *COX-2, NF-*κ*B*, and *iNOS* was downregulated, while the mRNA expression of *c-Kit, SCF, eNOS* was upregulated in the small intestinal tissue of BC01. In addition, the expression levels of these genes in the high-dose group were higher than in the low-dose group. Overall, the mRNA expression results indicated that administration of BC01 into the constipated mice could affect the expression of constipation-related genes in the small intestines of the mice.

**Table 2:** The levels of mRNA expression.

	***COX-2***	***NF-κB***	***iNOS***	***eNOS***	***c-Kit***	***SCF***
NC	1.00 ± 0.07^b^	1.08 ± 0.50^a^	1.00 ± 0.12^a^	1.00 ± 0.08^c^	1.00 ± 0.11^d^	1.00 ± 0.06^d^
M	5.06 ± 0.35^d^	2.85 ± 2.42^a^	6.57 ± 2.53^c^	0.39 ± 0.15^a^	0.36 ± 0.03^a^	0.31 ± 0.01^a^
DC	0.43 ± 0.05^a^	2.21 ± 0.84^a^	1.89 ± 1.17^ab^	0.52 ± 0.05^b^	0.53 ± 0.06^b^	0.58 ± 0.04^c^
BC01-H	0.41 ± 0.02^a^	1.80 ± 0.20^a^	3.26 ± 0.56^b^	0.51 ± 0.04^b^	0.65 ± 0.05^c^	0.63 ± 0.04^c^
BC01-L	1.42 ± 0.31^c^	1.47 ± 1.93^a^	3.43 ± 1.57^b^	0.44 ± 0.03^ab^	0.42 ± 0.04^a^	0.49 ± 0.03^b^

NC, normal group; M, constipation model group; DC, bisacodyl control group; BC01-H, B. coagulans BC01 high-dose group; BC01-L, B. coagulans BC01 low-dose group (*n* = 6); COX-2, Cyclooxygenase-2; NF-κB, Nuclear Factor-κB; iNOS, inducible nitric oxide synthase; eNOS, endothelial nitric oxide synthase; c-Kit, CD117; SCF, stem cell factor. ^a−d^Means in the same column with different lowercase superscript letters are significantly different at *P* < 0.05.

### Effects of BC01 on expression of inflammation-related proteins

**Figure 5:**
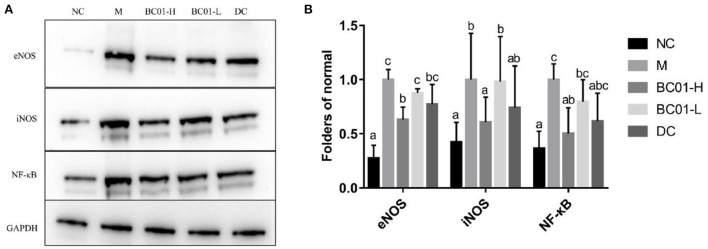
Protein expression in the small intestinal tissue. **(A)** Representative Western-Blot for the small intestinal tissue. **(B)** Western-Blot quantifications. The different letters among the experimental groups indicated statistical differences (*p* < 0.05). NC, normal group; M, constipation model group; DC, bisacodyl control group; BC01-H, *B. coagulans* BC01 high-dose group; BC01-L, *B. coagulans* BC01 low-dose group.

The effect of BC01 on the expression levels of inflammation-related proteins in small intestine tissue is shown in Figure 5. The protein expression levels of the inflammatory factors eNOS, iNOS, and NF-κB in each treatment group were higher in the M group, followed by DC/BC01-L, BC01-H, and then NC. Therefore, compared to the NC group, the expressions of these inflammatory proteins were significantly upregulated in the M group. However, after the intervention of BC01 and bisacodyl, the expressions of these inflammatory proteins was downregulated, with the degree of downregulation being the most significant in the BC01-H group.

### Fecal microbial diversity

**Figure 6:**
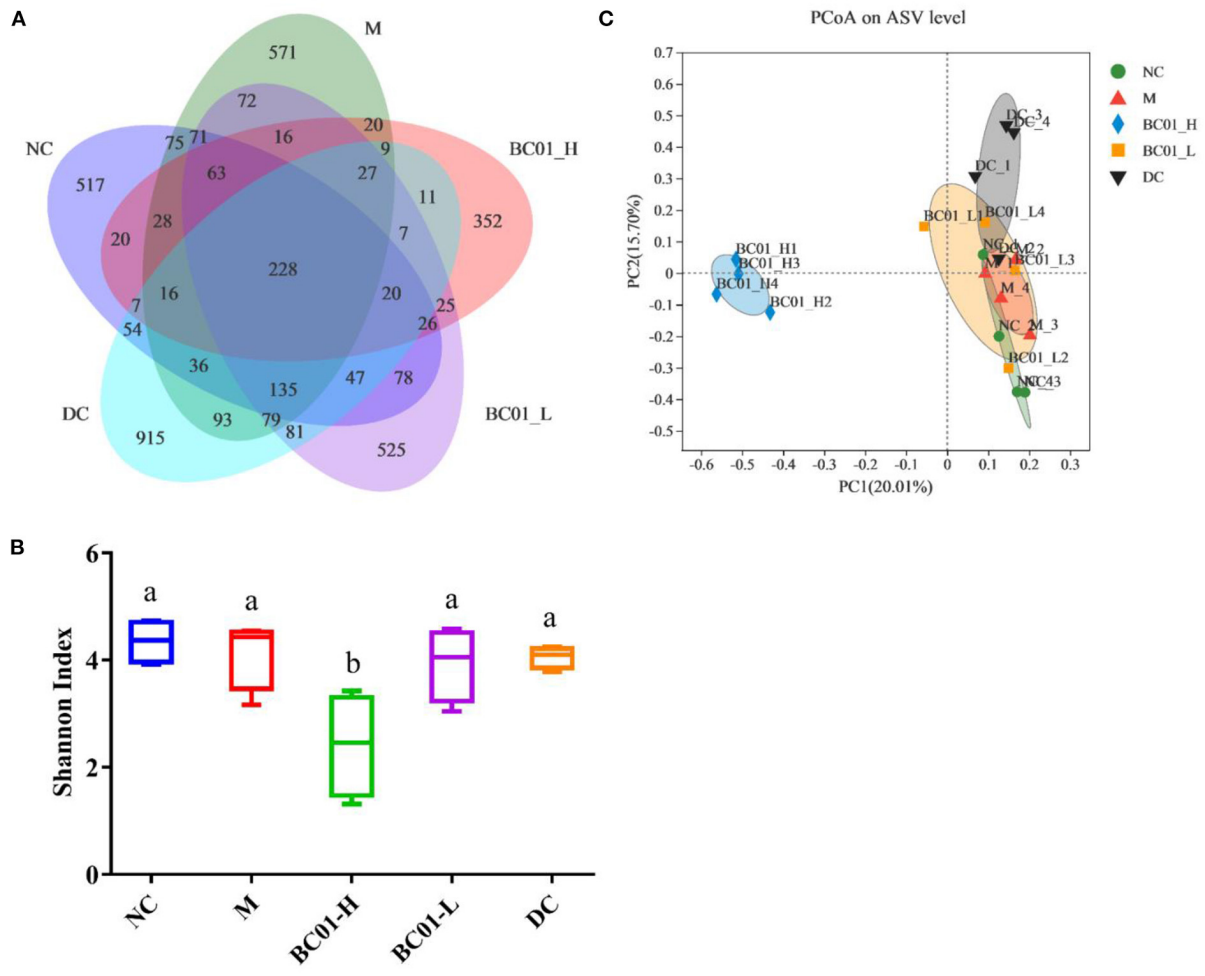
Effects of BC01 on the intestinal microbiota composition in the constipated mice. **(A)** Venn plot of shared and unique ASVs in mouse gut microbiota. **(B)** Shannon index of the ASV level. **(C)** ASV level principal co-ordinates analysis using Bray-Curtis metrics. NC, normal group; M, constipation model group; DC, bisacodyl control group; BC01-H, *B. coagulans* BC01 high-dose group; BC01-L, *B. coagulans* BC01 low-dose group (*n* = 4).

Sparse curves are mainly used to determine alpha diversity of intestinal microbiota. The sparse curve results in Supplementary Figure S1 showed that the number of species detected in the sample increased greatly with the sequencing depth. After the sequencing amount increased to 10,000, while the species did not increase significantly, it also did not reach saturation. The current sequencing data and depth were sufficient to reflect the composition of most of the microbiota in the above samples. A Venn diagram was used to visualize the community composition of the M and the sample groups and clearly illustrate the effect of BC01 on the composition and richness of intestinal microbiota in the constipation model mice (Figure 6A). The result showed the number of bacterial ASVs shared and unique to the five groups. There were 228 shared ASVs in the five sample groups. The NC group had 517 unique ASVs, the M group had 571 unique ASVs, the BC01-H had 352 unique ASVs, the BC01-L had 525 unique ASVs, and the DC had 915 unique ASVs.

The Shannon index is a diversity index that is used to represent the diversity of bacterial communities in a sample. The larger the Shannon index value, the higher the community diversity. The microbial community species diversity (α diversity) in the samples was estimated based on the Shannon index (Figure 6B). The BC01-H group had the lowest community diversity (*p* < 0.05 vs. other groups). However, the community diversity of BC01-L was not significantly different from other groups, indicating that high doses of *Bacillus* had a significant effect on community diversity (*p* < 0.05). Based on the bray_curtis distance algorithm, the β diversity analysis was conducted through a principal component analysis (PCoA) of the differences in bacterial community structure between the groups at the ASV level. The PCoA results (Figure 6C) showed that the PC1 could explain 20.01% of the data, while the PC2 could explain 15.70%. On the PC1 and PC2 axes, the sample points corresponding to the intestinal microbiota in the BC01-H group were significantly separated from those in the M group, and the community structure was significantly different.

### Comparison of microbial diversity between groups

**Figure 7:**
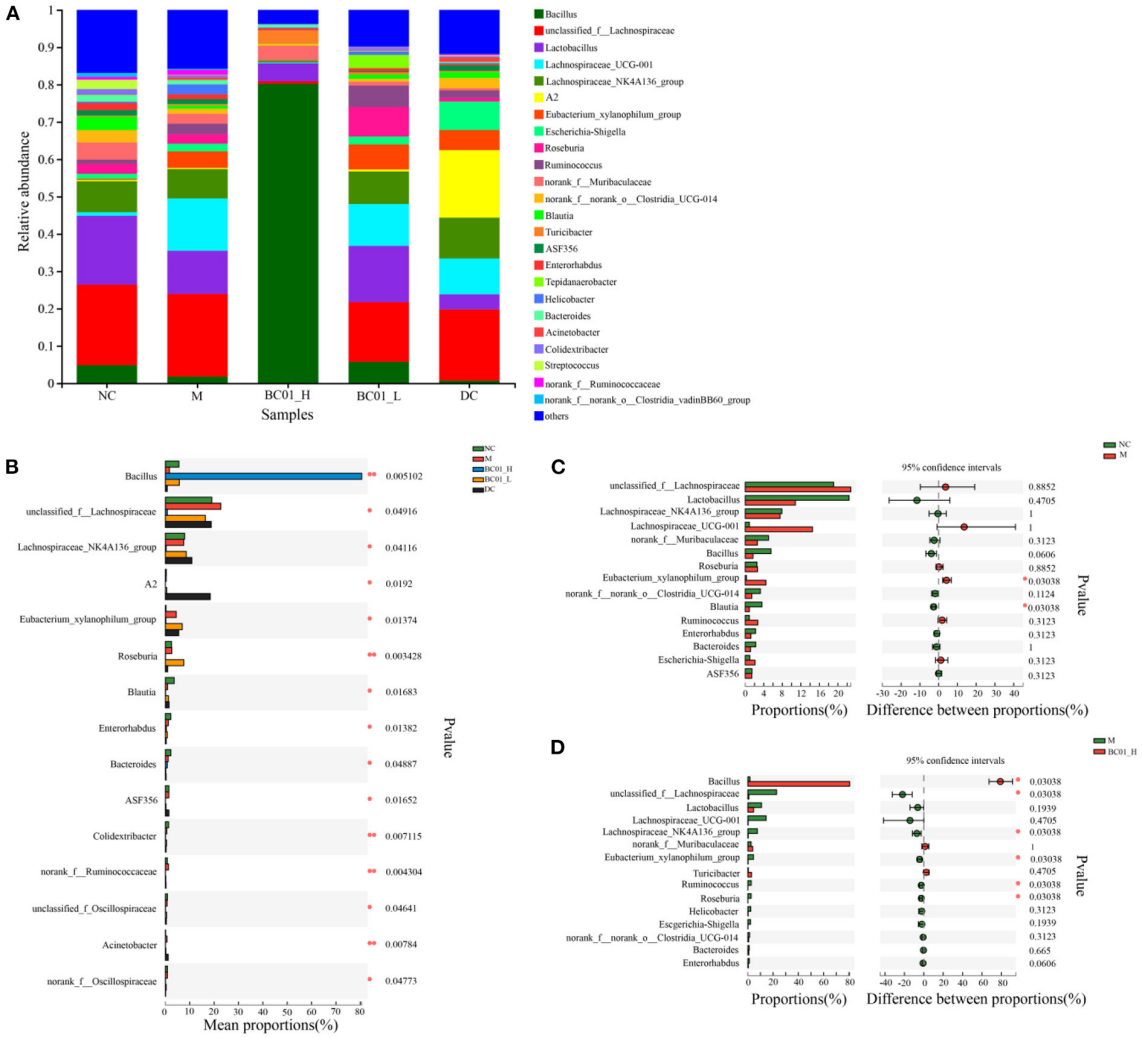
Effects of BC01 on the intestinal microbial composition and structure in the constipated mice. **(A)** Changes in the relative abundance of gut microbiota at the genus level. **(B)** Comparison of the gut microbiota composition between the experimental groups at the genus level. **(C)** Microflora comparison between the NC and M groups at the genus level. **(D)** Microflora comparison between the M and BC01-H groups at genus level. The *p* values are shown on the right. NC, normal group; M, constipation model group; DC, bisacodyl control group; BC01-H, *B. coagulans* BC01 high-dose group; BC01-L, *B. coagulans* BC01 low-dose group (*n* = 4).

We compared and analyzed the compositional changes of the gut microbial communities among the experimental groups at genus level. At genus level, the gut microbiota structure and relative abundance of bacterial species in the five groups of mice are shown in Figure 7A. In the fecal samples, *unclassified_f_Lachnospiraceae, Lactobacillus, Lachnospiraceae_UCG-001, Lachnospiraceae_NK4A136_group* and *Eubacterium_xylanophilum_group* had higher abundances in the M group, accounting for 22.25%, 11.56%, 13.40%, 7.85%, and 4.44%, respectively. After the BC01 intervention, *Unclassified_f_Lachnospiraceae, Lachnospiraceae_UCG-001, Lachnospiraceae_NK4A136_group*, and *Eubacterium_xylanophilum_group* relative abundances decreased to 0.68, 0.24, 0.33, and 0.20%, respectively. However, the relative abundances of *Bacillus* and *Lactobacillus* increased from 1.78 to 80.22% and from 11.56 to 15.05%, respectively. BC01 supplementation also improved the abundances of other genera. Interestingly, the microbiota in the BC01-H group is dominated by the probiotic strain, the main genus is *Bacillus*. Species level analysis was performed on the BC01-H group, and *Bacillus* accounted for 80.22% (Supplementary Figure S2), including *Unclassified_g_Bacillus* (46.65%), *Bacillus_thermolactis* (32.76%), and *Lactobacillus_vaginalis_g_Bacillus* (0.81%).

Analysis of species abundance between groups of sample microbial communities using the between-group difference test. Based on the proportion of the sequence, the Kruskal-Wallis H test was used for difference analysis. The results showed that there were significant differences in microbial communities among the groups (Figure 7B). *Bacillus, unclassified_f_Lachnospiraceae, Lachnospiraceae_NK4A136_group, A2, Eubacterium_ xylanophilum_group, Roseburia, Blautia, Enterorhabdus, Bacteroides* have significant differences (*p* < 0.05). A Wilcoxon rank sum test was used to analyze the difference between the two groups. As shown in Figure 7C, the proportion of *Eubacterium_xylanophilum_group* in the M group was significantly higher than that in the NC group, while the proportion of *Blautia* in the NC group was significantly higher than that in the M group (*p* < 0.05). After BC01 treatment, the proportion of Bacillus in the BC01-H group was significantly higher than that in the M group. The proportion of *unclassified_f_Lachnospiraceae, Lachnospiraceae_NK4A136_ group, Eubacterium_xylanophilum_group, Ruminococcus*, and *Roseburia* in the M group was significantly higher than that in the BC01-H group (*p* < 0.05) (Figure 7D).

## Discussion

Constipation is a common gastrointestinal disorder worldwide. Probiotics have been shown to have great potential for treating constipation (18, 19). In particular, *B. coagulans*, which has the ability to form spores and has tolerance to heat, oxygen, dry, acid and bile acids, has attracted extensive attention (20). Several clinical studies in recent years have shown that *B. coagulans* has a beneficial effect in the treatment of constipation. Using *B. coagulans* Unique IS2 supplemented with 2 billion CFU once daily for 4 weeks, the probiotic group had significantly more bowel movements compared to the placebo group (*p* < 0.001). 98% of subjects achieved normal stool consistency and relief of incomplete bowel movements, painful bowel movements, and abdominal pain associated with constipation (21). Lactulose is an osmotic laxative that relieves constipation by increasing the amount of fluid in the intestines and softening the stool (22). The addition of *B. coagulans* Unique IS2 to lactulose reduced the time required to relieve constipation compared to lactulose alone (23). The results of a 2-week clinical trial with 20 volunteers taking *B. coagulans* SANK 70258 (1 × 10^8^ CFU/d) isolated from green malt showed that intake of SANK 70258 improved colonic transit time, intestinal environment, bowel frequency, and fecal characteristics of the subjects (20). In addition, *B. coagulans* LBSC could up-regulate *Actinobacteria* and *Firmicutes* in the human body, down-regulate *Bacteroides, Proteobacteria, Streptophyta*, and *Verrucomicrobia*, change various metabolic pathways related to microbiota, and create a normal intestinal microenvironment (24). Potential mechanisms of *B. coagulans* on constipation may include altering intestinal microbiota, improving colonic transport time, or lowering the pH of the intestinal environment due to the production of short-chain fatty acids (25), thus exerting beneficial effects.

In this study, we used loperamide hydrochloride to establish a functional constipation model in mice. Loperamide hydrochloride is a peripheral opioid receptor agonist that can induce constipation symptoms by inhibiting water secretion, reducing stool weight and water content, inhibiting intestinal peristalsis, and reducing intestinal transport capacity, thereby reducing the number of fecal particles in the intestines and prolonging the defecation time (15, 26). After modeling with loperamide hydrochloride, the fecal water content and intestinal propulsion rate were reduced in the M of mice, compared with NC. After treatment, the fecal water content of the mice increased and the intestinal propulsion rate was significantly increased (*p* < 0.05). The small intestine propulsion rate mainly reflects the propulsion ability of the small intestine part. The higher the small intestine advancement rate, the stronger the small intestine peristalsis. The results indicate that BC01 has the ability to increase fecal moistening, accelerate intestinal peristalsis and improve small intestinal propulsion rate, thus relieving constipation. This is consistent with the findings of Tang et al. (27), among others.

The integrity of the small intestinal villi is a key factor in evaluating constipation severity. Damage to small intestinal villi will also affect the intestinal peristalsis function to varying degrees. H & E staining showed that the intestinal tissue structure was abnormal in the M group. The number of villi in some areas of the mucosal layer was reduced, epithelial cells were eroded and shed, and inflammatory cells and fibrous tissue proliferation were seen. After BC01 treatment, the tissue structure of the small intestine was basically normal, and no obvious inflammatory cell infiltration was seen.

Altering the homeostasis of the intestinal microbiota is a critical factor in inducing constipation (5). The intestinal microbiota forms a type of intestinal barrier, the biological barrier, by adhering or binding to the intestinal mucosa (28). The biological barrier is involved in regulating the intestinal immune system, resisting the invasion and injury of pathogenic bacteria and maintaining the ecological balance of the microbiota. Compared with M, after BC01 treatment, the dominant bacteria at the family level are *Bacillus* family, and the dominant bacteria at the genus level are *Bacillus* spp. and *Turicibacter*. This is consistent with the results of previous clinical studies (13, 29). In addition, *Roseburia* was also increased, and *Roseburia* accelerates intestinal transport (30). In summary, when orally delivered, BC01 can effectively improve the dysbiosis in constipation by enriching *Bacillus* and beneficial bacteria, and has shown laxative effects. Probiotics improve constipation by increasing the richness and diversity of the intestinal microbiota, changing the composition of the microbiota and the production of metabolites, acting on the nervous and immune systems, etc., and regulating intestinal motility.

Intestinal nerve parameters that promote intestinal motility and the transport of contents are secreted by intestinal neural networks in the gastrointestinal tract (31). Gastrointestinal hormones are small-molecule peptides, some of which also exist in the central nervous system, so they are also referred to as brain-gut peptides or neurotransmitters (32–34). After binding to receptors on target cells, gastrointestinal hormones play important regulatory roles in the absorption, movement, secretion, and immune health of the digestive system through different signaling mechanisms, some of which are closely related to constipation (35). In this study, we investigated the effect of BC01 supplementation of the levels of various gastrointestinal hormones, including MTL, ET-1, SS, VIP, and SP, in the model mice. Intestinal defecation requires the participation and regulation of smooth muscle and the nervous system, and the inhibitory brain-gut peptide SS regulates the contractile function of gastrointestinal smooth muscle. SS secretion promotes the secretion of the vasoactive peptide VIP and neurotransmitter SP, leading to smooth muscle relaxation, which in turn affects defecation (36). SP is a neurotransmitter distributed throughout nerve fibers that stimulates sensation and movement in the gut (37). ET-1 is mainly produced in vascular endothelial cells and is involved in vasoconstriction; high concentrations can cause gastrointestinal motility disorders (34). Increasing the levels of SP and MTL can accelerate intestinal peristalsis, improve transport rate and improve constipation symptoms, while VIP, SS, and ET-1 are important factors in slowing down intestinal transit time, and reducing their secretion can relieve constipation (38). Riezzo G et al. (39) measured serum MTL and SS levels to investigate whether there were pathophysiological differences between healthy controls and patients with slow-transit and normal-transit constipation, and showed that patients with slow-transit constipation had lower MTL levels and higher SS levels. Liu Shan (40) found that compared with normal healthy mice, the serum levels of MTL and SP were significantly lower and the SS levels were significantly higher (*p* < 0.01) in the mice of the constipation model group, the intragastric administration of Yangyin tonMi capsule can significantly regulate the level of the above-related gastrointestinal peptides close to that of normal mice, so as to alleviate the symptoms related to constipation. This study found that SP levels were lower in the constipated mice compared to the control mice, while the levels of SS, VIP, and ET-1 were higher, resulting in a decrease in intestinal motility. BC01 treatment significantly increased the serum SP and MTL levels but reduced serum ET-1, SS, and VIP levels, and the high BC01 dose had a more prominent effect than the low dose.

BC01 also affected the mRNA expression of various genes implicated in constipation. Interstitial cells of Cajal (ICC) are involved in promoting intestinal peristalsis (41). Several studies have shown that ICC was lower in patients with chronic constipation, which was mainly caused by the downregulation of *c-Kit* expression (42, 43). The *c-Kit* (CD117) is a transmembrane protein with tyrosine kinase activity, also known as stem cell factor receptor, and is a specific marker of ICC (44). *SCF* is the ligand of *c-Kit*, and the proliferation, differentiation, and phenotype maintenance of ICC are affected by changes in the secretion and expression of *SCF/c-Kit* (45). Compared to the NC group, M group had lower *c-Kit* mRNA levels, but the *SCF/c-Kit* ratio was significantly increased after BC01 treatment. Loperamide hydrochloride can cause the transcription level of *c-Kit* and *SCF* to be significantly lower than that of NC, while BC01 can restore the transcription level after intragastric administration. The results shown that BC01 may alleviate constipation by increasing the transcription level of *c-Kit* and *SCF*, which is consistent with previous studies (46). In addition, the mRNA expression levels of inflammation-related proteins, including NF-κB, iNOS, and eNOS, were lower in the colon tissue of the mice treated with BC01 compared to the control mice.

In summary, BC01 can achieve constipation relief by regulating intestinal gene transcription levels, intestinal fluid secretion and smooth muscle contraction, alleviating intestinal inflammation and restoring intestinal microbiota homeostasis, and then repairing the chemical and biological barriers, respectively. It is thus speculated that the pathway of BC01 effect on relieving constipation may be that the intervention leads to changes in the abundance of relevant microorganisms, thus altering the metabolites of the microbiota or activating signaling pathways to achieve the repair of intestinal barrier function and effectively improve constipation symptoms.

## Conclusion

*B. coagulans* BC01 was found to increase fecal water content, promote intestinal peristalsis, and improve the propulsion rate in the small intestine of constipated mice. In addition, our results also showed that *B. coagulans* BC01 could alter intestinal function by regulating gastrointestinal motility, gene expression of gastrointestinal-related proteins, intestinal inflammation, and the homeostasis of the intestinal microbiota. These results described herein suggested that Probiotic *B. coagulans* BC01 has the ability to prevent and treat constipation.

## Data availability statement

The original contributions presented in the study are included in the article/Supplementary materials, further inquiries can be directed to the corresponding author.

## Ethics statement

The animal study was reviewed and approved by the Institutional Animal Care and Use Committee of Southwest University of China, IACUC-20211020-06.

## Author contributions

XZ: provided the conception, conceived the study, and writing—review and editing. YC: data curation and software. XM and XY: visualization and investigation. YY: supervision. XC: writing—review and editing. HS: conceptualization, funding acquisition, and supervision. All authors contributed to the article and approved the submitted version.

## Funding

This study was supported by Innovation Center of Food Safety and Quality Control in Jiangsu Province; University Innovation Research Group in Chongqing (CXQT21007) and Key Construction Disciplines of Traditional Chinese Medicine in Chongqing (2021-4322190044).

## Conflict of interest

Authors XM, YY, and XY were employed by Thankcome Biological Science and Technology (Su Zhou) Co., Ltd. The remaining authors declare that the research was conducted in the absence of any commercial or financial relationships that could be construed as a potential conflict of interest.

## Publisher's note

All claims expressed in this article are solely those of the authors and do not necessarily represent those of their affiliated organizations, or those of the publisher, the editors and the reviewers. Any product that may be evaluated in this article, or claim that may be made by its manufacturer, is not guaranteed or endorsed by the publisher.
