# Supplementary material for: Efficacy of Bacillus coagulans BC01 on loperamide hydrochloride-induced constipation model in Kunming mice
Source: Front Nutr. 2022 Sep 21;9:964257. doi: 10.3389/fnut.2022.964257 (PMC9533339; doi:10.3389/fnut.2022.964257)
Supplement: Supplementary file 1 [file Data_Sheet_1.zip › Supplementary materials/Figure S1 and Figure S2.docx]

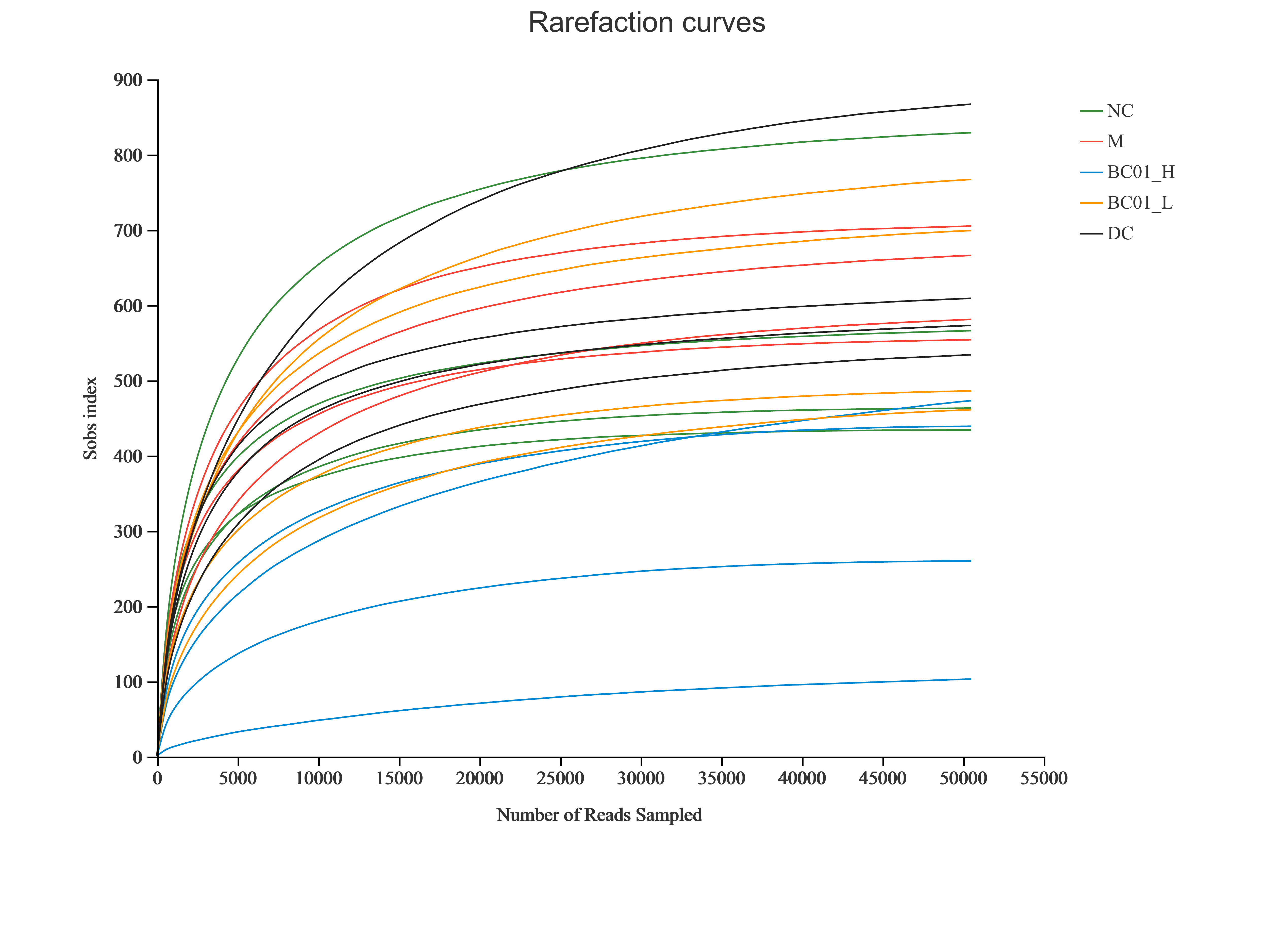


Figure S1. Sample ASV rarefaction curve.


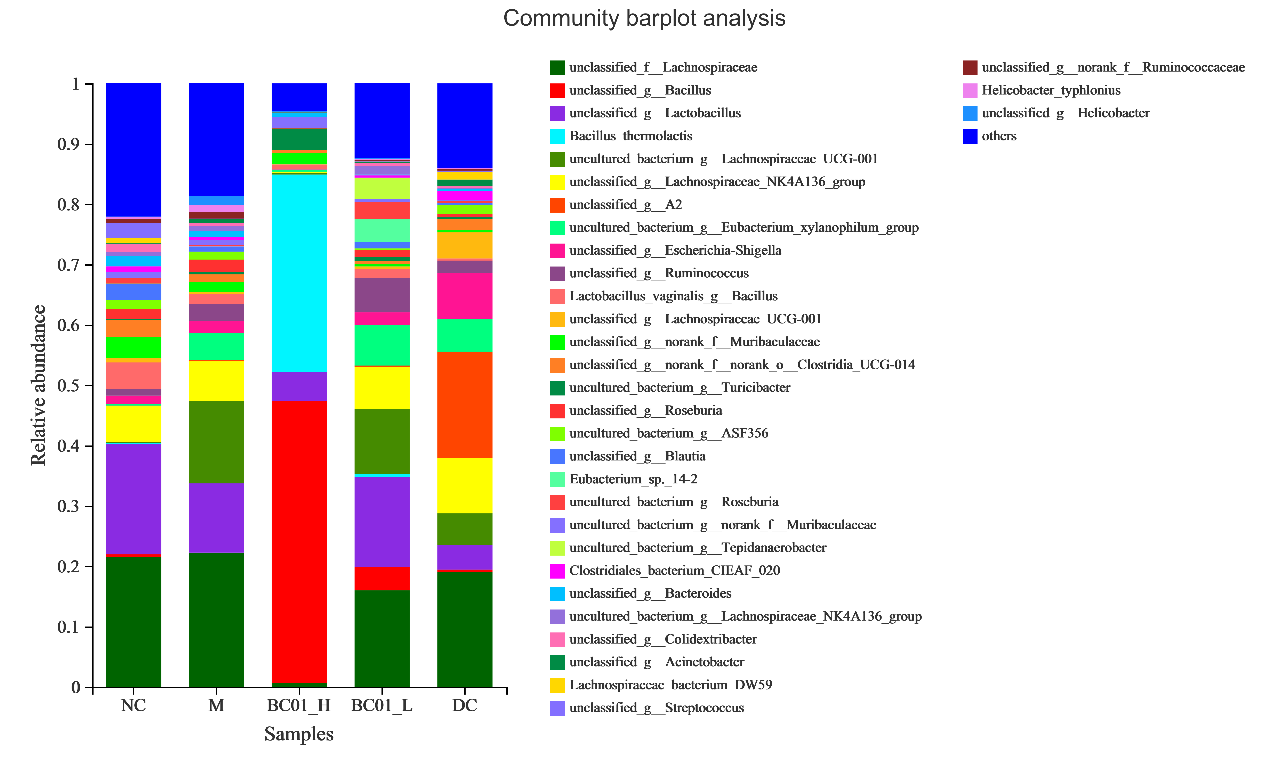


Figure S2. Changes in the relative abundance of gut microbiota at the species level.
